# Evolving concepts of tumor heterogeneity

**DOI:** 10.1186/2045-3701-4-69

**Published:** 2014-11-25

**Authors:** Victoria R Zellmer, Siyuan Zhang

**Affiliations:** Department of Biological Science, Harper Cancer Research Institute, University of Notre Dame, A130 Harper Hall, Notre Dame, IN 46556 USA

**Keywords:** Tumor heterogeneity, Tumor evolution, Tumor microenvironment, Cancer stem cell, Omic analysis and personalized therapy

## Abstract

Past and recent findings on tumor heterogeneity have led clinicians and researchers to broadly define cancer development as an evolving process. This evolutionary model of tumorigenesis has largely been shaped by seminal reports of fitness-promoting mutations conferring a malignant cellular phenotype. Despite the major clinical and intellectual advances that have resulted from studying heritable heterogeneity, it has long been overlooked that compositional tumor heterogeneity and tumor microenvironment (TME)-induced selection pressures drive tumor evolution, significantly contributing to tumor development and outcomes of clinical cancer treatment. In this review, we seek to summarize major milestones in tumor evolution, identify key aspects of tumor heterogeneity in a TME-dependent evolutionary context, and provide insights on the clinical challenges facing researchers and clinicians alike.

## Introduction

Cancer has been traditionally typified by a stepwise accumulation of mutations in key oncogenes and tumor suppressors [1]. For decades, accumulation of these traits in somatic cells has been considered as the foundation of a developmental model of tumor progression where cells transition from a normal, healthy state to pre-malignant, malignant, and migratory phenotypes [1]. Consequently, tumors display distinguishing traits, defined as hallmarks of cancer [2], that demarcate malignant cells from normal cells [3].

Meanwhile, tumors are often described as heterogeneous, owing to the intricate genetic diversity and assorted morphological phenotypes they embody [2]. Intratumor heterogeneity specifically refers to heterogeneity within a tumor, while intertumor heterogeneity refers to heterogeneity across several different tumors [3]. The current view of tumor heterogeneity recognizes basic principles of Darwinian evolution at the core of neoplastic development and outgrowth: a single somatic cell with a heritable fitness-promoting mutation proliferates, conferring a survival advantage that allows cells to outlast the less ‘fit’ cells [3, 4]. Natural selection leads to sequential waves of clonal expansion, resulting in various subclones with differing capacities for proliferation, migration, and invasion [5]. While similarities promoting tumor survival are maintained among subclones, changes in the local tumor microenvironment (TME) further influence genetic divergence and phenotypic outcomes [5]. This rigorous fitness test promotes genomic instability, thus contributing to the vast heterogeneity observed in cancer genomes [2, 6, 7].

Advances in next-generation sequencing techniques and the inception of The Cancer Genome Atlas (TCGA) have revealed extensive heterogeneity at the molecular level [8]. However, scientists and physicians remain perplexed by the origins of cancer heterogeneity and its mechanistic and clinical implications. Understanding tumor heterogeneity is the first of many important steps toward improving both the clinical management and treatment of cancer.

In this review, we will revisit the key milestones in tumor evolution, highlight the evolving concepts of tumor heterogeneity, and provide insight on the clinical challenges facing researchers and clinicians alike.

## Major milestones in tumor evolution

Three hundred years after the invention of the microscope, concurrent with the dawn of Darwinian evolution, German physiologist Johannes Muller and his assistants applied microscopy to human tumor samples in 1833. Until this point, all recorded knowledge of tumors was collected with the naked eye, leaving layers of critical information untapped. Applying methods used by botanists and plant physiologists, Muller transformed pathology and modern medicine with his monograph on cancer. This led to his conjecture that tumors are composed of new cells within a diseased organ. Muller and colleagues morphologically distinguished carcinoma subtypes within a single tumor and noted variation among tumor-adjacent connective tissues, detailing the vast heterogeneity observed. It was Muller’s student, famed pathologist Rudolf Virchow, who later determined that all tumors derive from normal cells. Muller and Virchow transformed modern medicine not just by inventing the field of pathology, but also by recording some of the earliest evidence that tumors are heterogeneous [9, 10].

**Figure 1:**
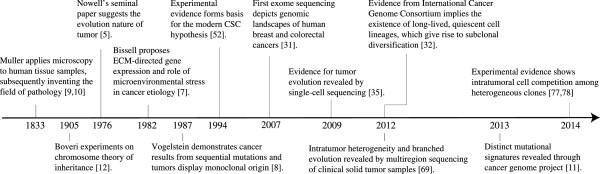
**Timeline of the evolving concepts of tumor heterogeneity.**

All tumors possess some form of somatic mutation, and our current understanding of tumor heterogeneity is built upon the principle that acquired mutations are heritable [11]. Essential to this point is Theodor Boveri’s keen observation at the beginning of the twentieth century that aberrant mitoses are associated with malignant tumors and his findings on inheritance factors [12]. Boveri traced the fate of each cell and found that cells with different chromosome combinations were phenotypically dissimilar, which led to two main conclusions: (1) chromosomes transmit different inheritance factors and (2) unequal chromosome distribution is detrimental to normal development [13]. Decades later, key reports by David Hungerford, Peter Nowell, and Janet Rowley further substantiated Boveri’s hypothesis, becoming one of the most important milestones in cancer research [14, 15]. In 1976, Nowell published a now infamous paper depicting a working model for tumor evolution [5]. Among several persuasive thoughts, Nowell described a cancer progression model where major genetic errors drive natural selection of cells with improved fitness in response to intrinsic and extrinsic pressures. This ecological view of tumor development has captivated researchers and become a core concept in today’s cancer research (Figure 1).

## Classic view: heritable tumor heterogeneity

### Genetic heterogeneity

Genetic heterogeneity of tumors is rooted in one of the key hallmarks of cancer: genetic instability [2]. Several mechanisms are in place in normal cells that protect against chromosome and nucleotide damage by preventing DNA replication until damage is repaired; however, genes controlling these critical checkpoints (e.g. p53) are often perturbed in cancer cells [16]. Genetic instability in cancer has been demonstrated at both the nucleotide level in point mutations and chromosome level in translocations, deletions, amplifications, and complete chromosome aneuploidy [17]. One of the major genetic dysfunctions initiating cancer is telomere crisis, which is characterized by extensive cell death and concomitant cytogenetic abnormalities [18]. Telomere crisis results in end-to-end chromosome fusion passed on to daughter cells that subsequently harbor the same chromosome rearrangement patterns and dsDNA fracture [19]. It is surmised that cancer initiation progresses toward malignancy once the fittest clone survives extreme chromosomal rearrangement events in the absence of protective telomeres [20]. As this cell population expands, negative selection occurs against clones with detrimental rearrangements. In many circumstances, it is probable that a multitude of cells survive, each with a unique genome, resulting in a high degree of intratumor genetic heterogeneity [3].

**Figure 2:**
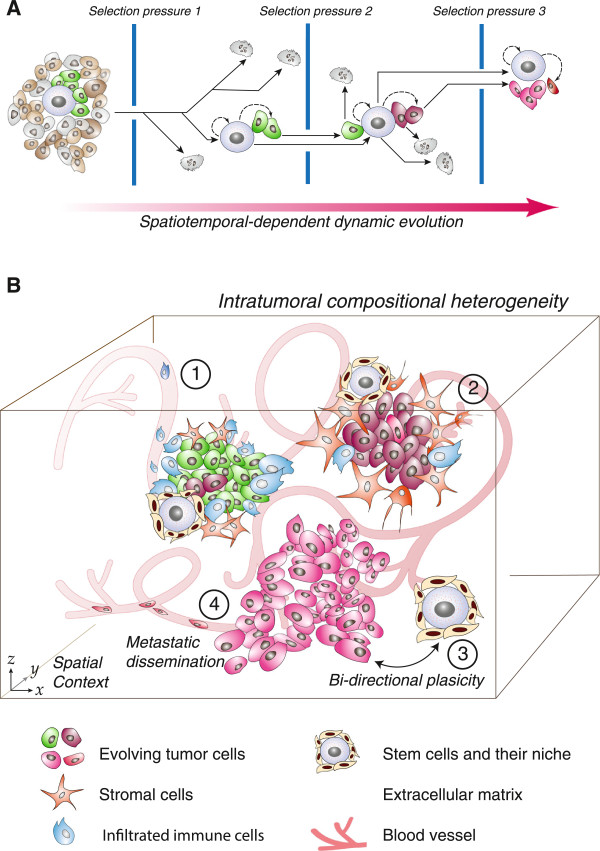
**Tumor evolution and compositional heterogeneity. A**, Evolution drives heritable heterogeneity and subsequent outgrowth of malignant clones. Selection pressures from the local microenvironment (e.g. hypoxia, secretion of growth-inhibiting factors, chemotherapeutic agents, etc.) challenge tumor cell survival, often resulting in cell death in early cancer initiation. In order to survive these in a given tissue niche, cancer cells must acquire mutations that promote survival and tumor formation with regard to spatiotemporal context. Robust cells capable of surviving multiple selection events acquire proliferative advantages, eventually resulting in tumor progression and evidence of genetic heterogeneity within a tumor. **B**, Snapshots of natural selection events within the TME paint a heterogeneous portrait of tumor composition in a spatial context. The TME refers to both the tumor and its local environment of diverse resident and migratory cell types. 1) Infiltrated immune cells shape the tumor development; 2) Tumor stromal cells interact with tumor cells and change the local cancer stem cell niche; 3) Bi-directional plasticity between stem-like cancer cells and tumor cells. 4) Disseminated tumor cells.

Tumor cells undergo a series of genetic events that contribute to genomic instability throughout tumor progression (Figure 2A). However, the specific mechanisms and precise order in which they occur have yet to be elucidated [21]. Studies have pursued these mechanisms and found that the rate at which mutations occur in somatic cells is insufficient to cause the striking number of mutations present in cancer genomes. Over the past few decades, a ‘mutator’ hypothesis tumor evolution has emerged, speculating that a mutator phenotype characterized by genomic instability drives multi-step carcinogenesis and explaining the mutation rate discrepancy observed in normal and malignant cells [22]. This concept was initially described in Nowell’s paper where he attributes the high number of mutations in cancer genomes to waves of clonal selection [5, 23]. Studies in bacteria and yeast imply mutator mutations confer a selective growth advantage on cells harboring these acquired mutations [24, 25]. The current mutator hypothesis speculates that a small number of ‘driver’ alterations exist and, once acquired by somatic mutation, confer the cancer phenotype; however, seemingly insignificant ‘passenger’ mutations result via mechanisms yet to be elucidated [26]. McFarland *et al.* challenged this with stochastic simulation of tumor evolution and reasoned that, though individually weak, the cooperative burden of small-scale accumulated passenger mutations has a present role in tumor progression, and may be the cause for complex oncological events that remain unanswered by the driver-centric model [27].

Genomic analyses have provided evidence that drastic rearrangement events such as aneuploidy, a defining feature of genetic instability and cancer, and chromothripsis drive cancer progression [28]. Despite relative detection ease, the precise evolutionary advantage of aneuploidy in cancer progression remains unclear. Studies in *C. albicans* suggest aneuploidy promotes fitness throughout drug resistance evolution, similar to cancer, contrasting *S. cerevisiae*, which displays growth deficits as a result of aneuploidy [29]. Others have reported that *S. cerevisiae* diploids exhibit an increased number aneuploidy events under strong selection pressure [30]. Comparable to yeast, it is possible that aneuploidy occurs when survival is most threatened and the need to employ a rapid rearrangement mechanism is highest.

In the past 10 years, genetic sequencing data from independent laboratories and collective efforts from The Cancer Genome Atlas (TCGA) and ICGC (International Cancer Genome Consortium) produced global genetic profiles of different types of cancer [31–36]. As depicted in Figure 1, these milestones studies provides a new framework for future omic analyses based personalized cancer therapy.

### Epigenetic heterogeneity

The epigenome is defined as the whole suite of epigenetic factors that regulate expression of the genome and includes both heritable and non-heritable cellular changes that have been shown to contribute to tumor development and progression [37]. Temporal and spatial gene regulation has been recently appreciated in cancer biology. By far the most intensively studied heritable epigenetic alteration is DNA methylation, pioneered by Feinberg and Vogelstein [38]. Decreased methylation of satellite DNA has been associated with abnormal chromosome rearrangement and aneuploidy [38, 39]. Furthermore, tumor-specific aberrations in DNA methylation of tumor suppressor promoter regions have been well characterized [40].

Next generation sequencing techniques have advanced the current understanding of the epigenome and further complicated the current concept of tumor heterogeneity. Chromatin immunoprecipitation followed by sequencing (ChIP-seq) offers single nucleotide resolution, an unlimited dynamic range, and the capacity to multiplex samples [41]. Recently, ChIP-seq has been implemented in the identification of long-range epigenetic activation (LREA) in DNA regions containing microRNAs, oncogenes, and cancer biomarker genes, where Bert *et al.* found that epigenetic alterations can be influenced by adjacent genes [42]. Another recent study by Vanharanta *et al.* applied ChIP-seq to show epigenetic enabling of the von Hippel-Lindau (VHL) tumor suppressor activation of hypoxia inducible factors (HIFs) for metastasis [43]. More over, Hansen *et al.* recently reported an increased variance of putative CpG sites in tumor cells compared to normal cells across several types of cancer [44]. Significant loss of methylation stability block regions of DNA implied that tumor heterogeneity might potentially evolved from loss of epigenetic stability of well-defined genomic domains [44].

## Tumor heterogeneity: the origin of tumor species

Cancer evolution and heterogeneity is a long debated subject that questions the tumor origin. Borrowing principles of evolution and biodiversity, scientists have reasoned that tumors originate in stem cell populations, as the innate longevity of stem cells increases the chance of acquiring harmful mutations [45]. Increasing evidence from studies on hematopoietic cancers [46], breast cancers [47], and brain cancers [48] has led researchers to believe that cellular heterogeneity of the tumor has been largely attributed to clonal expansion of putative cancer stem cells (CSCs). The CSC model addresses two key components of tumorigenesis: tumor origin and tumor capacity. CSCs are defined as cells that can both self-renew and give rise to the various cell types within a tumor [49]. Central to this hypothesis is the notion that tumors originate in tissue stem cells (i.e. a particular progenitor population within the tissue) as a result of disordered self-renewal mechanisms [45]. Accordingly, tumor cells display a hierarchical order of potential in which cells of the highest order possess self-renewal and simultaneous multi-lineage differentiation capacity [50].

Early clonogenic and tumor sphere forming assays showed evidence of stem like cells in heterogeneous tumors; however, these *in vitro* assays are not a true assessment of self-renewal capacity [45]. Further confirmation of a CSC population was the clinical observation that certain leukemia displayed poorly proliferative progenitor population [51]. Moreover, John Dick and colleagues performed groundbreaking studies that led to the identification and proposition of a CSC population in acute myeloid leukemia (AML) [52]. Lapidot *et al.* isolated the reputed CSC population using classical stem cell markers from patient peripheral blood and demonstrated that a subpopulation of progenitor cells could recapitulate AML in SCID mice and displayed potential for self-renewal. These findings formed the basis for the modern CSC hypothesis and led to the further identification of cancer stem-like cells tumor initiating cells in breast cancer and brain tumors [45].

The traditional CSC hypothesis implies that cellular hierarchies exist in tissues with stem cells (in normal tissues) or CSC (in tumors) at their respective apices [53]. Chaffer and colleagues challenged the traditional CSC hypothesis in their demonstration that human mammary epithelial cells can revert to a stem-like state under certain conditions rather than adhering to unidirectional differentiation hierarchy [54]. This study and others, while extensively debated, characterize the dynamic phenotypic changes tumor cells undergo to promote survival, migration, and proliferation at secondary sites [55]. Transient phenotypic shifts such as the epithelial-mesenchymal (EMT) and mesenchymal-epithelial transitions (MET), are understood as conversions facilitating cell plasticity, but have recently gained appreciation as events underlying compositional tumor heterogeneity (Figure 2B) in unison with the findings discussed below by Wang and colleagues [56] and Chaffer and colleagues [53].

## Tumor microenvironment-driven transient compositional tumor heterogeneity

It is abundantly clear that the evolutionary selection of fit clones is a system-wide process that occurs in a dynamic tissue milieu termed the tumor microenvironment (TME) [57]. Bissell and colleagues pioneered the concept [58] that a progressively remodeled TME influences both genetic and compositional heterogeneity [59]. Increasing evidence demonstrates that changes in the tumor ecosystem drive compositional tumor heterogeneity. Hoadley *et al.* compiled an extensive molecular taxonomy report across several different cancer types where tissue of origin provided the strongest identification signal [60]. This key result is not surprising, as epithelial-adjacent stroma could differ from connective, nervous, and muscular stroma. A study by Wang *et al.* provides direct evidence that the tumor stroma harbors a deregulated ECM that promotes malignancy and intratumoral heterogeneity in mammary gland models [56]. Michor and Weaver claim these findings as further evidence of neo-Darwinian evolution in cancer [61]. These reports question the current tumor cell centric model of plasticity by implying cancer cells posses a dynamic, almost sentient nature.

### TME-imposed heterogeneity derives from CSCs

Stem cell self-renewal and differentiation is dictated by the microenvironment, or stem cell niche. Normal stem cell niches are generally located in hypoxic tissue niches (e.g. mammary stem cells in the basal compartment of the mammary gland) that promote the stem cell phenotype. Poorly vascularized tumors contain hypoxic regions with undifferentiated ‘stem-like’ tumor cells that survive under control of HIFs [62]. Yeung *et al.* used 3D cell culture to demonstrate that hypoxia inhibits differentiation of colon cancer cells and maintains a stem-like phenotype [63]. In addition, the putative stem cell niche constitutes numerous cross-talking stromal cells. Vermeulen *et al.* demonstrated that myofibroblasts secrete factors that maintain the CSC population in colon cancer cell culture models [64]. They showed stromal cells impose a CSC phenotype on differentiated cancer cells, justifying the transient morphological heterogeneity observed in cancer. Recently, Chaffer *et al.* reported basal breast cancers cells retain the ZEB1 promoter in a configuration allowing ample response to environmental signals [53]. These results corroborate a cancer cell plasticity model where conversions occur between various cell states with fluctuating tumorigenic capacities. These recent findings, in summary, mark the beginning of a momentous conceptual shift in the CSC hypothesis and tumor evolution.

### Infiltrated stromal cells and tumor heterogeneity

Studies on deregulation of the tumor secretome provide compelling evidence for the TME as a major contributor to compositional tumor heterogeneity. Substantial evidence supporting a role for inflammation in cancer progression has been reported in the last decade and is commonly accepted as a hallmark characteristic of the TME [65]. One of the major mechanisms of tumorigenesis is production of self-sustaining inflammatory cytokines (e.g. IL-1, IL-6, TNF) by pre-malignant cells, resulting in extensive recruitment of diverse immune cells and challenging cellular fitness by altering niche dynamics [66]. Constitutive activation of NF-κB impedes activity of the tumor suppressor p53, a prominent hub in DNA-induced cellular stress networks and regulator of cellular senescence [67]. Reciprocally, tumor development conditions the surrounding TME. Lujambio *et al.* recently demonstrated that, in the context of chronic liver inflammation, depletion of a p53-dependent senescence program in tumor cells results in increased cirrhosis and fibrosis that promotes adjacent epithelial malignant transformation and transient intratumoral heterogeneity [68].

The collective interplay between the CSCs and the TME results in compositional intratumor heterogeneity (Figure 2B). However, the in-depth molecular mechanisms of this dynamic interplay along with functional consequences of compositional heterogeneity have yet to be revealed.

### Final thoughts: the ugly truth of tumor heterogeneity

The dawning of the age of ‘omics’ brought with it great hope for discovery and validation of novel biomarkers, relevant drug targets, and disease-specific signatures [69]. Powerful sequencing technologies have painted a daunting portrait of tumor evolution [11, 32, 34, 70] and tumor heterogeneity [69]. Genomic heterogeneity is not the only hurdle to overcome: recent advances in single cell RNA-seq also depicted epigenetically regulated transcriptome heterogeneity in primary glioblastoma [71]. The current paradigm of personalized medicine involves tailoring therapy around profiled signaling variations between tumors [72]. With significant strides made in understanding tumor heterogeneity in recent years, it is unsettling that the conventional treatment strategy is to profile the tumor based on the most prevalent clone at the time of diagnosis or relapse, ignoring the ugly truth that intratumoral heterogeneity promotes the evolutionary nature of tumor development.

Careful consideration of the complete tumor context is essential to understanding and developing more effective personalized treatments that address tumor heterogeneity. The first challenge is whether genetic and compositional profiling of multifocal tumors of monoclonal origin displaying intrafocal heterogeneity can be effectively manage [73]. Multifocality occurs in 30% of breast cancer cases and 50-76% of prostate cancer cases, among others [74, 75]. Current reports suggest directing treatment at the dominant foci largely underestimates malignant potential, further highlighting the need to better understand each patient’s genetic and compositional tumor heterogeneity [75]. To tackle this challenge, Fujii *et al.* recently generated a computer model to further study multifocal prostate cancer based on data obtained from 152 human prostatectomy specimens evaluated by DNA microarray analysis, where they demonstrated heterogeneous individual foci with a common clonal precursor [76]. Beckman *et al.* reported another mathematical model of personalized treatment that integrates dynamics of evolutionary genetics into analysis and treatment design. Their analyses of hypothetical cases as well as a simulated clinical trial of over 3 million qualified ‘patients’ showed that augmented and, occasionally counterintuitive, nonstandard treatment strategies may lead to improved patient survival compared with the current model of personalized medicine [72].

A number of new concepts have emerged in recent years. The concept of intratumoral cell competition among heterogeneous clones reshaped our classic hierarchical view of heterogeneity and potentially can be exploited as therapeutic entry points in eradicating multifocal cancers [77]. Cleary *et al.* showed evidence of two genetically distinct tumor cell subclones in communication to maintain the tumor population [78]. This leading-edge report sheds light on how diverse tumor cell populations persist despite clonal selection, often thwarting current clinical therapies. In addition, many studies have explored how heterogeneity within a specific type of cancer promotes self-seeding and metastatic outgrowth. Campbell *et al.* employed advanced genomics techniques to further understand the underlying mechanisms driving pancreatic cancer progression and metastasis. Despite showing vast genetic diversity, the authors were able to elucidate a distinct pattern of genomic instability [79]. Moreover, to better stratify the clinical cancer subtypes, Gatza *et al.* recently used integrated genomics to characterize the functional role of key genetic driver mutations in luminal breast cancer and correlated specific genetic signatures with poor prognosis [80]. Although the genetic diversity reported in findings is daunting, these results represent tremendous strides forward for potential identification of therapeutic targets for diseases with few clinical options.

With massive omics data generated from The Cancer Genome Atlas (TCGA), various algorithms and tools for recognition of activated and altered pathways exist for integrative analysis of two or more types of omics data and are rapidly proving worthwhile [81]. Notably, Kristensen *et al.* used Pathway Recognition Algorithm using Data Integration on Genomic Models (PARADIGM) analyses based on copy number alterations (CNAs) and mRNA expression of data from the MicroMetastases Project (MicMa) cohort to show that integrated analysis of DNA copy number alteration and mRNA expression leads to improved prognostic discrimination of patients compared to separate analysis of any other molecular levels [82]. Five distinct clusters of invasive breast cancer were identified and found to uniformly express a chronic inflammatory signature [82]. Similarly, The Physical Sciences-Oncology Network completed another project implementing integrated omics analyses to characterize basic breast cancer research models. Over twenty labs designed a series of multidisciplinary comparative studies on two cell lines: MCF10A (non-tumorigenic breast cells) and metastatic breast cancer cells (MDA-MB-231 cell line). Comprehensive network signatures for motility, morphology, and cellular stress were constructed from transcriptomics and proteomics data from each cell line [83]. Among their results was the finding that integrin-β4 is a common node between the non-tumorigenic and metastatic breast cancer cell lines [83].

## Conclusions

Taken together, future integrated omics analyses with consideration of compositional heterogeneity inferred by interplay between intratumoral subclones and TME will allow us to identify more robust biomarkers and devise therapeutic strategies for cancer treatment, such as staggering targeted therapies to keep selection pressures minimal [84]. Mapping the evolutionary roots of tumor heterogeneity will be the foundation of personalized medicine in the oncology clinic in the foreseeable future.

## Abbreviations

TME: Tumor microenvironment
TCGA: The Cancer genome atlas
ICGC: International cancer genome consortium
ChIP-seq: Chromatin immunoprecipitation followed by sequencing
VHL: von Hippel-Lindau
HIF: Hypoxia inducible factors
CSC: Cancer stem cell
AML: Acute myeloid leukemia
SCID: Severe combined immunodeficient
EMT: Epithelial-mesenchymal transition
MET: Mesenchymal-epithelial transition
ZEB1: Zinc finger E-box binding homeobox 1
NF-κB: Nuclear factor kappa-light-chain-enhancer of activated B cells
PARADIGM: Pathway recognition algorithm using data integration on genomic models
CNA: Copy number alteration
MicMa: MicroMetastases Project
dsDNA: Double-stranded DNA.
